# Development and validation of an explainable machine learning prediction model of hemorrhagic transformation after intravenous thrombolysis in stroke

**DOI:** 10.3389/fneur.2024.1446250

**Published:** 2025-01-15

**Authors:** Yanan Lin, Yan Li, Yayin Luo, Jie Han

**Affiliations:** ^1^Department of Neurology, The First Affiliated Hospital of Dalian Medical University, Dalian, China; ^2^Interdisciplinary Research Center for Biology and Chemistry, Liaoning Normal University, Dalian, China

**Keywords:** acute ischemic stroke, intravenous thrombolysis, hemorrhagic transformation, machine learning, explainability

## Abstract

**Objective:**

To develop and validate an explainable machine learning (ML) model predicting the risk of hemorrhagic transformation (HT) after intravenous thrombolysis.

**Methods:**

We retrospectively enrolled patients who received intravenous tissue plasminogen activator (IV-tPA) thrombolysis within 4.5 h after symptom onset to form the original modeling cohort. HT was defined as any hemorrhage on head CT scan completed within 48 h after IV-tPA administration. We utilized the Random Forest (RF), Multilayer Perceptron (MLP), Adaptive Boosting (AdaBoost), and Gaussian Naive Bayes (GauNB) algorithms to develop ML-HT models. The models' predictive performance was evaluated using confusion matrix (including accuracy, precision, recall, and F1 score), and discriminative analysis (area under the receiver-operating-characteristic curve, ROC-AUC) in the original cohort, followed by validation in an independent external cohort. The models' explainability was assessed using SHapley Additive exPlanations (SHAP) global feature plot, SHAP Summary Plot, and Partial Dependence Plot.

**Results:**

A total of 1,007 patients were included in the original modeling cohort, with an HT incidence of 8.94%. The RF-based ML-HT model showed metrics of 0.874 (accuracy), 0.972 (precision), 0.890 (recall), 0.929 (F1 score); with ROC-AUC of 0.7847 in the original cohort and 0.7119 in the external validation cohort. The MLP model showed 0.878, 0.967, 0.989, 0.978, 0.7710, and 0.6768, respectively. The AdaBoost model showed 0.907, 0.967, 0.989, 0.978, 0.7798, and 0.6606, respectively. The GauNB model showed 0.848, 0.983, 0.598, 0.716, 0.6953, and 0.6289, respectively. The explainable analysis of the RF-based ML model indicated that the National Institute of Health Stroke Scale (NIHSS) score, age, platelet count, and atrial fibrillation were the primary determinants for HT following IV-tPA thrombolysis.

**Conclusion:**

The RF-based explainable ML model demonstrated promising predictive ability for estimating the risk of HT after IV-tPA thrombolysis and may have the potential to assist the clinical decision-making in emergency settings.

## 1 Introduction

Intravenous tissue plasminogen activator (IV-tPA) thrombolysis remains the most effective and evidence-based treatment for acute ischemic stroke (AIS) patients (1). Despite its proven efficacy, IV-tPA thrombolysis carries a significant risk of hemorrhagic transformation (HT). The National Institute of Neurological Disorders and Stroke (NINDS) study has shown that patients undergoing IV-tPA thrombolysis had a higher incidence of HT compared to the placebo group (2). HT not only exacerbates the pre-existing neurological damage but also delays the initiation of antiplatelet or anticoagulant medications. This can lead to a poorer prognosis and even an increased risk of death for AIS patients receiving IV-tPA thrombolysis (3). Given the absence of effective treatments to reverse hematoma expansion following HT, it is crucial to screen for and predict HT after IV-tPA thrombolysis in high-risk individuals.

Predictive models are essential tools in healthcare as they help in anticipating patient outcomes based on historical data and statistical algorithms. They can assist the clinicians in risk assessment and enable personalized treatment. To individually evaluate the safety of IV-tPA on an individual basis, several clinical studies have developed predictive scores for HT following IV-tPA thrombolysis. These models are among the earliest to be developed and validated, offering the advantages of simplicity and convenience. Some of these scores are still utilized to assist physicians in the decision-making processes to evaluate the safety of IV-tPA. These predictive scores include the HAT (Hemorrhage After Thrombolysis) score (4), the MSS (Multicenter Stroke Survey) score (5), the SEDAN [blood Sugar, Early infarct signs and (hyper) Dense cerebral artery sign, Age, NIHSS (National Institutes of Health Stroke Scale)] score (6), and the GRASPS [Glucose at presentation, Race (Asian), Age, Sex (male), systolic blood Pressure at presentation, and Severity of stroke at presentation (NIHSS)] score (7). These scores have been established using traditional statistical methods and possess several limitations. Firstly, these predictive scores can only predict simple linear relationships between variables. They are unable to handle complex non-linear relationships and high-dimensional data. Secondly, these scores lack inclusivity for data with large variations, resulting in weak generalization capabilities for completely new populations. Thirdly, they are sensitive to outliers and noise, which can lead to poor accuracy and stability. Lastly, most data within the scores require manual selection and processing, potentially introducing bias into the predictive outcomes. These limitations highlight the need for a more accurate prediction model to enhance the early identification of HT in AIS patients undergoing IV-tPA thrombolysis.

Machine learning (ML), a pivotal branch of artificial intelligence, has been applied in medical imaging diagnostics and has demonstrated superior predictive capabilities and stability (8, 9). Machine learning offers several advantages over traditional statistical methods, particularly in handling complex data patterns and high-dimensional datasets. Firstly, ML algorithms can automatically discover patterns in data without explicit programming. Secondly, they are capable of handling large datasets efficiently, a capability that is beyond the reach of traditional models. Thirdly, ML models can adapt and learn from new data, allowing them to improve over time. Additionally, they can better handle noise in the data compared to traditional models, which can be sensitive to outliers. Lastly, machine learning provides a wide range of algorithms, allowing for flexibility in selecting the right model for a specific task (10). By leveraging these strengths of ML, we have developed and validated an accurate and explainable predictive model for HT following IV-tPA thrombolysis using ML algorithm. We hope the ML-HT model can precisely identify patients at high risk for HT, thereby enabling personalized care and potentially enhancing the outcomes for AIS patients undergoing IV-tPA thrombolysis.

## 2 Materials and methods

### 2.1 Study participants

We retrospectively enrolled patients with AIS who received IV-tPA thrombolysis at the Stroke Center of the First Affiliated Hospital of Dalian Medical University from January 2010 to December 2022, forming the original modeling cohort. Additionally, we retrospective collected AIS patients who underwent IV-tPA thrombolysis at the Third People's Hospital of Dalian from January 2020 to December 2022, serving as the external validation cohort. For both the original modeling cohort and the external validation cohort, the inclusion criteria were as follows: (1) age ≥ 18 years; (2) fulfillment of diagnostic criteria for AIS (1); (3) treatment with IV-tPA thrombolysis within 4.5 h from symptom onset. The exclusion criteria were as follows: (1) patients who underwent endovascular therapy; (2) patients diagnosed with stroke mimics; (3) patients with missing data; (4) patients lost to follow-up. The study was conducted in compliance with the Declaration of Helsinki and approved by the Ethics Committee of the First Affiliated Hospital of Dalian Medical University (Approval Number: PJ-KS-KY-2023-08).

### 2.2 Data collection

Two trained neurologists collected the data. All data were collected prior to IV-tPA administration and served as predicted variables for the model development. These included: (1) demographic data: gender and age; (2) medical history data: hypertension, diabetes, atrial fibrillation, coronary disease, stroke, antiplatelet therapy, smoking, and alcohol consumption; (3) clinical data: baseline systolic blood pressure (SBP), diastolic blood pressure (DBP), onset-to-treatment time (OTT), baseline NIHSS score, total dose of tPA, tPA dose type (0.9 or 0.6 mg/kg); (4) laboratory data: white blood cell count (WBC), platelet count (PLT), activated partial thromboplastin time (APTT), thrombin time (TT), prothrombin time (PT), international normalized ratio (INR), fibrinogen (Fib), blood glucose (BG); (5) imaging data: the hyperdense middle cerebral artery sign (HMCAS) (11), the massive cerebral infarction (MCI) sign characterized by hypodensity involving more than 1/3 of the middle cerebral artery territory (6), and Alberta Stroke Program Early CT Score (ASPECTS) on the non-contrast head CT scan (12).

### 2.3 Definition of HT

HT was utilized as the objective variable, defined as any hemorrhage detected on the secondary head CT scan conducted within 48 h after IV-tPA thrombolytic treatment, according to the Heidelberg classification (13).

### 2.4 Statistical analysis

Statistical analyses were conducted using SPSS version 26.0. Continuous variables were presented as medians (interquartile ranges, IQRs), and Mann-Whitney test was used to compare between the groups. Categorical variables were presented as counts with percentages, and either Pearson's chi-square test or Fisher's exact test was used to compare between the groups. All statistical analyses were two-sided. A significance level was set at *P*-value < 0.05.

### 2.5 Machine learning modeling

All ML models were developed using scikit-learn package in Python version 3.0. Four ML algorithms were chosen for the modeling, including Random Forest (RF), Multilayer Perceptron (MLP), Adaptive Boosting (AdaBoost), and Gaussian Naive Bayes (GauNB). We conducted a comprehensive evaluation of ML models, addressing the issue of data imbalance by employing the normalization method. To ensure the robustness of our assessments, we employed stratified K-fold cross-validation. In this process, we randomly divided both the HT and non-HT samples into K equal parts. For each iteration of the K-fold cross-validation, we used (K-1) parts for both HT and non-HT samples as the training set, and the remaining single part of each category as the testing set. This validation was repeated K times, and the results from all iterations were synthesized to assess the performance of the ML model. In our study, the value of K was set to 10.

We evaluated the predictive performance of ML models using several methods. Firstly, we utilized a confusion matrix to calculate metrics such as accuracy, precision, recall, and the F1 score. Secondly, we conducted discriminative analysis using the area under the receiver operating characteristic curve (ROC-AUC). Thirdly, we compared the predictive performance of the ML models with traditional scoring models using ROC-AUC. Lastly, we performed external validation using the independent cohort, which was assessed with both a confusion matrix and ROC-AUC.

To analyze the explainability of the ML models, we employed SHapley Additive exPlanations (SHAP) Global Feature Importance Plot, SHAP Summary Plot, and Partial Dependence Plot. These were used to identify and visualize the effects of the predictive variables on the ML models.

## 3 Results

### 3.1 The baseline characteristics of AIS patients in original modeling cohort

**Figure 1:**
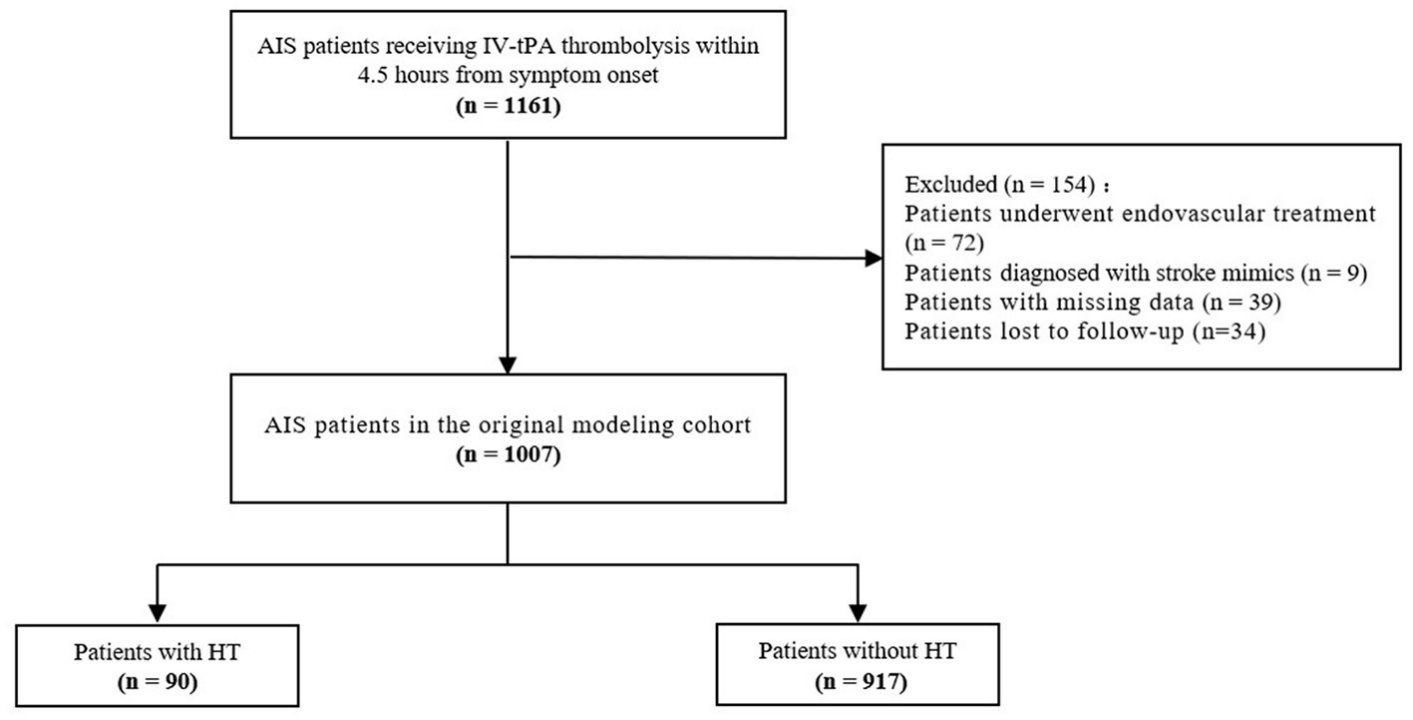
Flowchart of acute ischemic stroke patients selection in original modeling cohort. AIS, acute ischemic stroke; IV, intravenous; tPA, tissue plasminogen activator; HT, hemorrhagic transformation.

The study initially collected 1,161 AIS patients who received IV-tPA thrombolytic treatment within 4.5 h. After excluding 72 patients who underwent endovascular treatment, 9 patients diagnosed with stroke mimics, 39 patients with missing data, and 34 patients lost to follow-up, a total of 1,007 patients were ultimately included in the original modeling cohort (Figure 1). Patients had a median age of 67 years (IQR 59-73), and 60.4% were male. Among them, 90 patients developed HT (8.94%). Patients with HT showed statistically significant differences in terms of age, atrial fibrillation, NIHSS, WBC, PLT, HMCAS, and ASPECTS (*P* < 0.05) compared to patients without HT (Table 1).

**Table 1:** The baseline characteristics of AIS patients in original modeling cohort.

**Variables**	**Total AIS patients (*n* = 1,007)**	**Patients with HT (*n* = 90)**	**Patients without HT (*n* = 917)**	***Z*/χ^2^**	***P-*value**
**Demographic data**					
Male, *n* (%)	608 (60.4)	61 (67.8)	547 (59.7)	2.263	0.133
Age, years, median (IQR)	67 (59, 73)	72 (65, 80)	66 (59, 75)	4.533	0.000^*^
**Medical history data**, ***n*** **(%)**					
Hypertension	613 (60.9)	52 (57.8)	561 (61.2)	0.398	0.528
Diabetes	260 (25.8)	23 (25.6)	237 (25.8)	0.004	0.952
Atrial fibrillation	269 (26.7)	45 (50.0)	224 (24.4)	27.377	0.000^*^
Coronary disease	149 (14.8)	19 (21.1)	130 (14.2)	3.126	0.077
Stroke	242 (24.0)	22 (24.4)	220 (24.0)	0.009	0.924
Antiplatelet therapy	54 (5.4)	5 (5.6)	49 (5.3)	0.007	0.809
Smoking	345 (34.3)	28 (31.1)	317 (34.6)	0.435	0.509
Alcohol consumption	262 (26.0)	31 (27.4)	223 (26.1)	0.127	0.721
**Clinical data, median (IQR)**					
SBP, mmHg	154 (139, 169)	151 (136, 168)	154 (139, 170)	−0.715	0.474
DBP, mmHg	87 (78, 97)	85 (72, 95)	87 (79, 97)	−1.522	0.128
OTT, min	160 (120, 210)	180 (130, 226)	160 (120, 210)	1.919	0.055
NIHSS, score	7 (3, 13)	15 (9, 18)	7 (3, 12)	7.008	0.000^*^
tPA total dose, mg	60.0 (50.0, 67.5)	60.0 (50.0, 69.0)	60.0 (50.0, 67.5)	0.427	0.669
tPA dose type, *n* (%)				0.228	0.633
0.6 mg/kg	128 (12.7)	10 (11.1)	118 (12.9)		
0.9 mg/kg	879 (87.3)	80 (88.9)	799 (87.1)		
**Laboratory data, median (IQR)**					
WBC, 10^9^/L	7.33 (6.11, 9.28)	7.93 (6.45, 10.26)	7.31 (6.10, 9.16)	2.052	0.040^*^
PLT, 10^9^/L	201 (169, 240)	181 (151, 219)	204 (171, 241)	−3.958	0.000^*^
APTT, s	23.3 (21.1, 25.8)	23.5 (21.2, 25.8)	23.3 (21.1, 25.8)	0.385	0.700
TT, s	16.9 (15.8, 17.9)	17.0 (15.8, 17.8)	16.9 (15.8, 17.9)	0.697	0.485
PT, s	11.6 (11.0, 12.6)	11.7 (10.9, 12.8)	11.6 (11.0, 12.5)	0.641	0.522
INR	1.04 (0.97, 1.12)	1.05 (0.97, 1.15)	1.04 (0.97, 1.12)	1.059	0.290
Fib, g/L	2.66 (2.21, 3.22)	2.64 (2.21, 3.51)	2.66 (2.21, 3.21)	0.730	0.465
BG, mmol/L	6.95 (6.00, 8.96)	7.42 (6.22, 9.72)	6.93 (5.99, 8.90)	1.053	0.292
**Imaging data**, ***n*** **(%)**					
HMCAS	69 (6.9)	21 (23.3)	48 (5.2)	42.062	0.000^*^
MCI	17 (1.7)	2 (2.2)	15 (1.6)	0.170	0.659
ASPECTS, score, median (IQR)	10 (10, 10)	10 (10, 10)	10 (10, 10)	−4.960	0.000^*^

^*^P < 0.05.

### 3.2 Predictive performance of HT models based on different ML algorithms

HT prediction models were constructed using data from all 1,007 patients and 27 predictive variables. These models were developed using the RF, MLP, AdaBoost, and GauNB machine learning algorithms. After adjusting and optimizing the parameters for each algorithm, the predictive performance of the four ML-HT models was assessed using stratified K-fold cross-validation. This assessment included evaluation through confusion matrix, discriminative analysis, and external validation.

**Figure 2:**
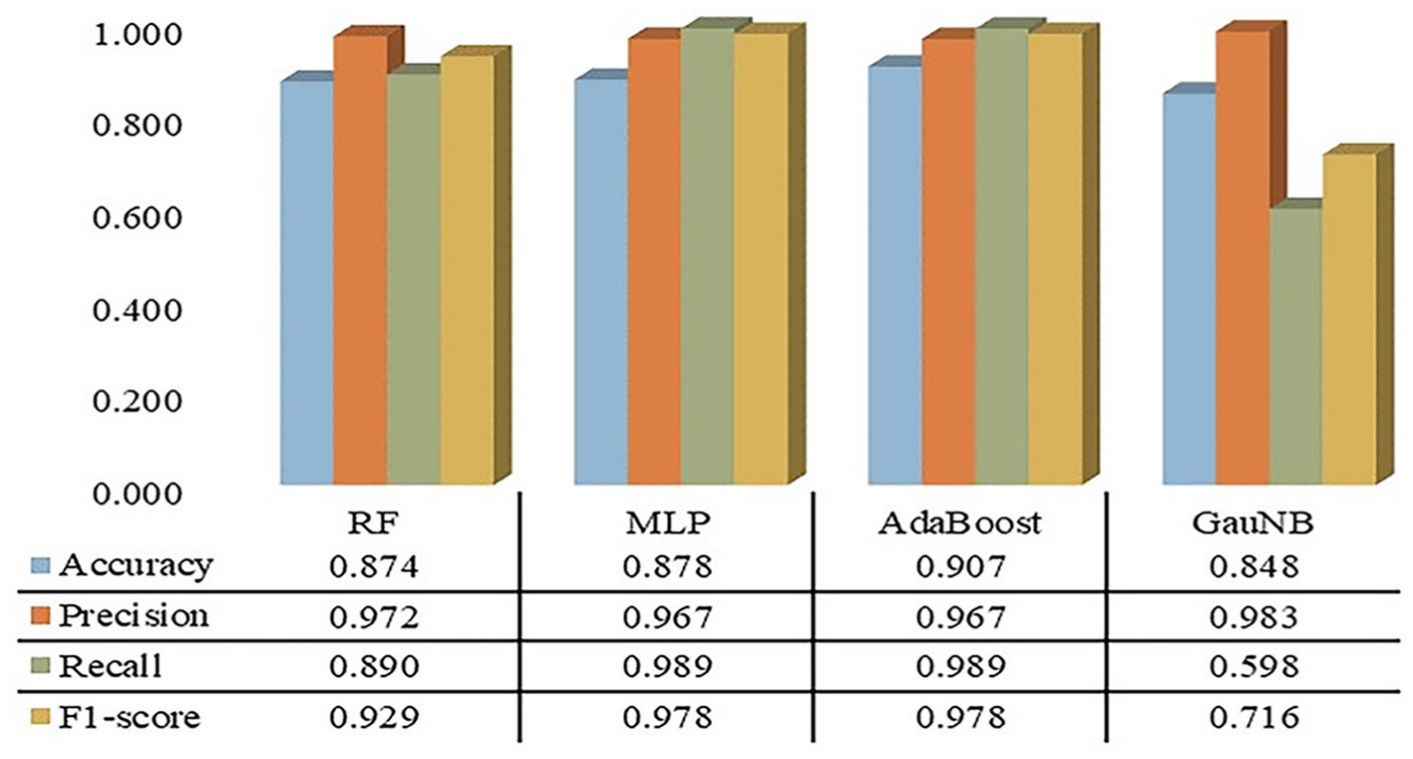
Confusion matrices for ML-HT prediction models based on RF, MLP, AdaBoost, and GauNB algorithms. This figure shows the confusion matrices for the ML-HT models based on RF, MLP, AdaBoost, and GauNB algorithms, which were generated using K-fold cross-validation. Confusion metrics including accuracy, precision, recall, and F1 score were calculated for each model. The ML-HT models based on RF, MLP, and AdaBoost demonstrate relatively stable performance, while the GauNB model exhibits weaker performance. ML, machine learning; HT, hemorrhagic transformation; RF, Random Forest; MLP, Multilayer Perceptron; AdaBoost, Adaptive Boosting; GauNB, Gaussian Naive Bayes.

The confusion matrices for the ML-HT models were derived through K-fold cross-validation, during which metrics such as accuracy, precision, recall, and F1 score were calculated. As shown in Figure 2, the average performance metrics for the ML-HT models based on four different algorithms were as follows: RF with accuracy: 0.874, precision: 0.972, recall: 0.890, and F1 score: 0.929; MLP with 0.878, 0.967, 0.989, and 0.978; AdaBoost with 0.907, 0.967, 0.989, and 0.978; and GauNB with 0.848, 0.983, 0.598, and 0.716. The ML-HT models based on RF, MLP, and AdaBoost algorithms were found to exhibit relatively favorable and stable performance in confusion matrix, in contrast to the GauNB, which showed weaker performance.

**Figure 3:**
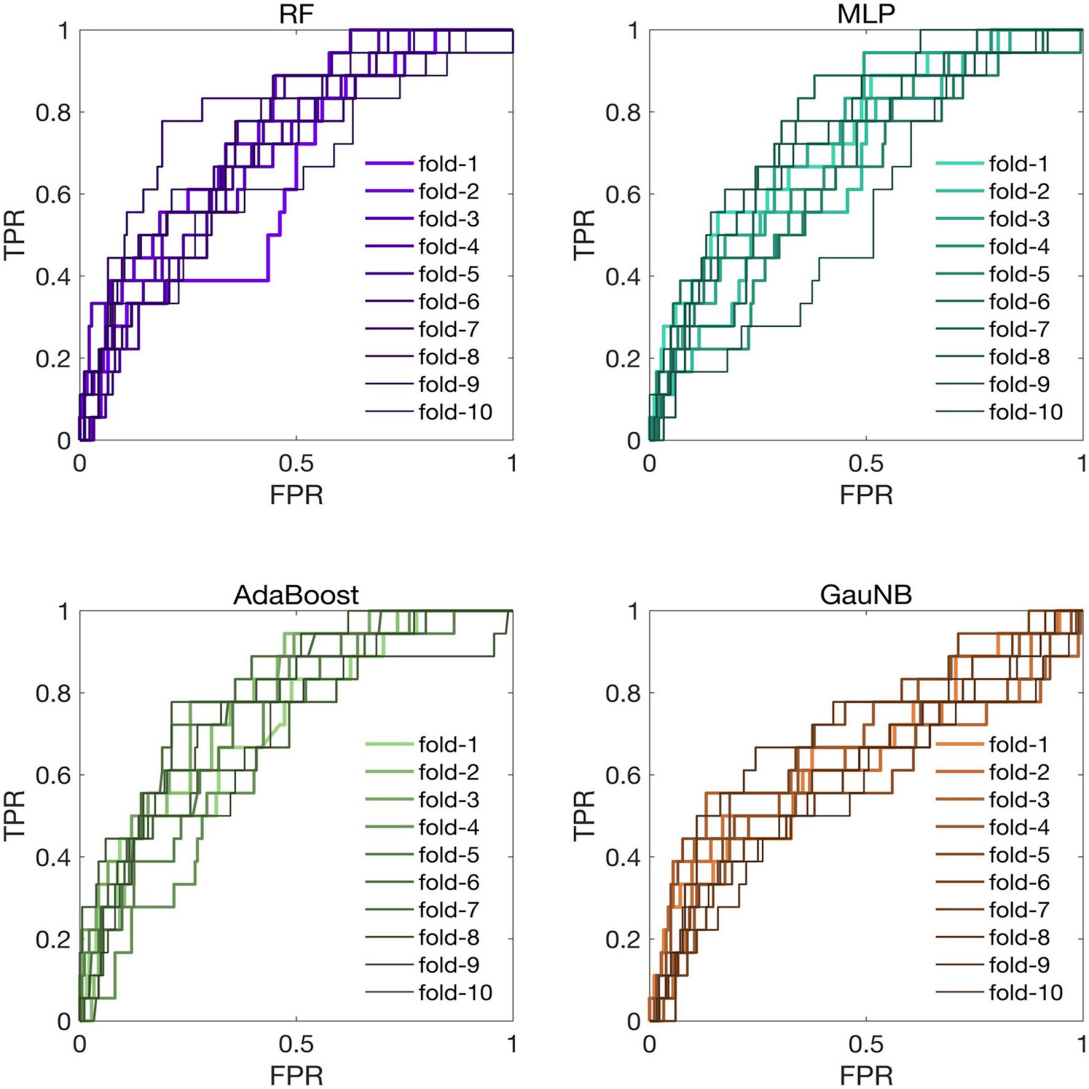
ROC curves for ML-HT prediction models based on RF, MLP, AdaBoost, and GauNB algorithms in the original cohort. This figure presents the ROC curves, which assess the discriminative ability of the ML-HT models validated in the original cohort. In K-fold cross-validation, the average ROC-AUC values for the ML-HT models based on RF, MLP, AdaBoost, and GauNB algorithms were 0.7847, 0.7710, 0.7798, and 0.6953, respectively. ROC, receiver operating characteristic; ROC-AUC, area under the ROC curve; ML, machine learning; HT, hemorrhagic transformation; RF, Random Forest; MLP, Multilayer Perceptron; AdaBoost, Adaptive Boosting; GauNB, Gaussian Naive Bayes; TPR, true positive rate; FPR, false positive rate.

The discriminative analysis for the ML-HT models was conducted on the original cohort using ROC curves. As depicted in Figure 3, the average ROC-AUC values from K-fold cross-validation for the ML-HT models based on RF, MLP, AdaBoost, and GauNB algorithms were 0.7847, 0.7710, 0.7798, and 0.6953, respectively. The ML-HT models constructed using RF, MLP, and AdaBoost algorithms exhibited favorable discrimination (ROC-AUC > 0.70), with the exception of the GauNB.

To compare the discrimination between the ML-HT models and the traditional predictive scores, we tested the ROC-AUC for the HAT, MSS, SEDAN, and GRASPS scores. The results showed that the predictive discrimination of different scores for HT after IV-tPA thrombolysis ranked from highest to lowest as follows: GRASPS, MSS, SEDAN, and HAT score, with corresponding ROC-AUC values of 0.712 (95% CI: 0.654–0.771), 0.684 (95% CI: 0.628–0.740), 0.653 (95% CI: 0.592–0.715), and 0.643 (95% CI: 0.579–0.707). Traditional statistical scores generally performed worse in predictive performance compared to the ML models, suggesting that their predictive capabilities are insufficient (Table 2).

**Table 2:** Discrimination validation of traditional predictive scores for HT.

**Scores**	**ROC-AUC (95% CI)**	**Cut-off**	**Sensitivity (%)**	**Specificity (%)**
GRASPS	0.796 (0.726–0.866)	81.5	77.4	75.1
MSS	0.724 (0.644–0.804)	1.5	71.0	64.3
SEDAN	0.715 (0.619–0.811)	1.5	60.2	65.7
HAT	0.714 (0.611–0.817)	1.5	45.2	89.1

**Figure 4:**
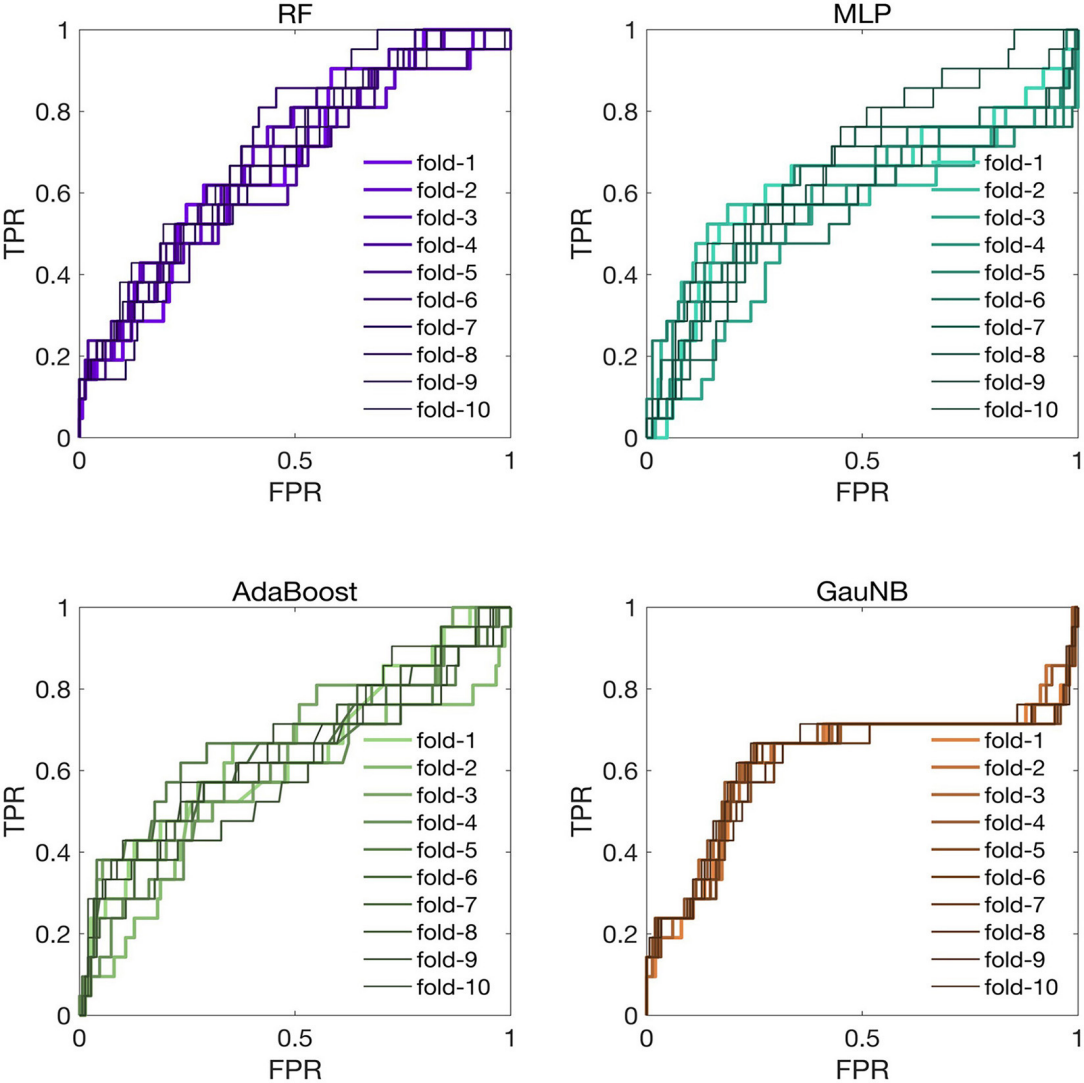
ROC curves for ML-HT prediction models based on RF, MLP, AdaBoost, and GauNB algorithms in the external validation cohort. This figure displays the ROC curves that assess the generalization of the ML-HT models as validated in an independent external cohort. In K-fold cross-validation, the average ROC-AUC values for the ML-HT models based on RF, MLP, AdaBoost, and GauNB were 0.7119, 0.6768, 0.6606, and 0.6289, respectively. ROC, receiver operating characteristic; ROC-AUC, area under the ROC curve; ML, machine learning; HT, hemorrhagic transformation; RF, Random Forest; MLP, Multilayer Perceptron; AdaBoost, Adaptive Boosting; GauNB, Gaussian Naive Bayes; TPR, true positive rate; FPR, false positive rate.

To assess the generalization of the ML-HT models, we conducted the external validation of their discriminative performance using an independent cohort from another stroke center. The ROC curves for the ML-HT models in the external validation cohort were presented in Figure 4. In K-fold cross-validation, the average ROC-AUC values for the ML-HT models based on RF, MLP, AdaBoost and GauNB were 0.7119, 0.6768, 0.6606, and 0.6289, respectively. Additionally, we tested the confusion matrices for the ML-HT models in the external cohort. The average performance metrics for the ML-HT models based on four different algorithms were as follows: RF with accuracy: 0.867, precision: 0.893, recall: 0.958, and F1 score: 0.929; MLP with 0.857, 0.884, 0.967, and 0.929; AdaBoost with 0.870, 0.882, 0.987, and 0.931; and GauNB with 0.816, 0.897, 0.886, and 0.896. Notably, only the ML-HT model using the RF algorithm demonstrated a more favorable discrimination and stable performance in confusion matrix in the external validation.

Taking into account the results from confusion matrices, discriminative analysis, and external cohort validation, the ML-HT model based on the RF algorithm emerged as the most stable with a favorable performance. This model's consistent high performance across different assessment methods suggests its robustness and potential reliability.

### 3.3 Explainable analysis of the ML-HT model based on RF algorithm

The ML-HT model based on RF algorithm, demonstrated relatively stable performance across the confusion matrix, discriminative analysis, and external validation assessment. Furthermore, we conducted an evaluation of its explainability to determine the impact of different predictive variables on the incidence of HT following IV-tPA thrombolysis.

**Figure 5:**
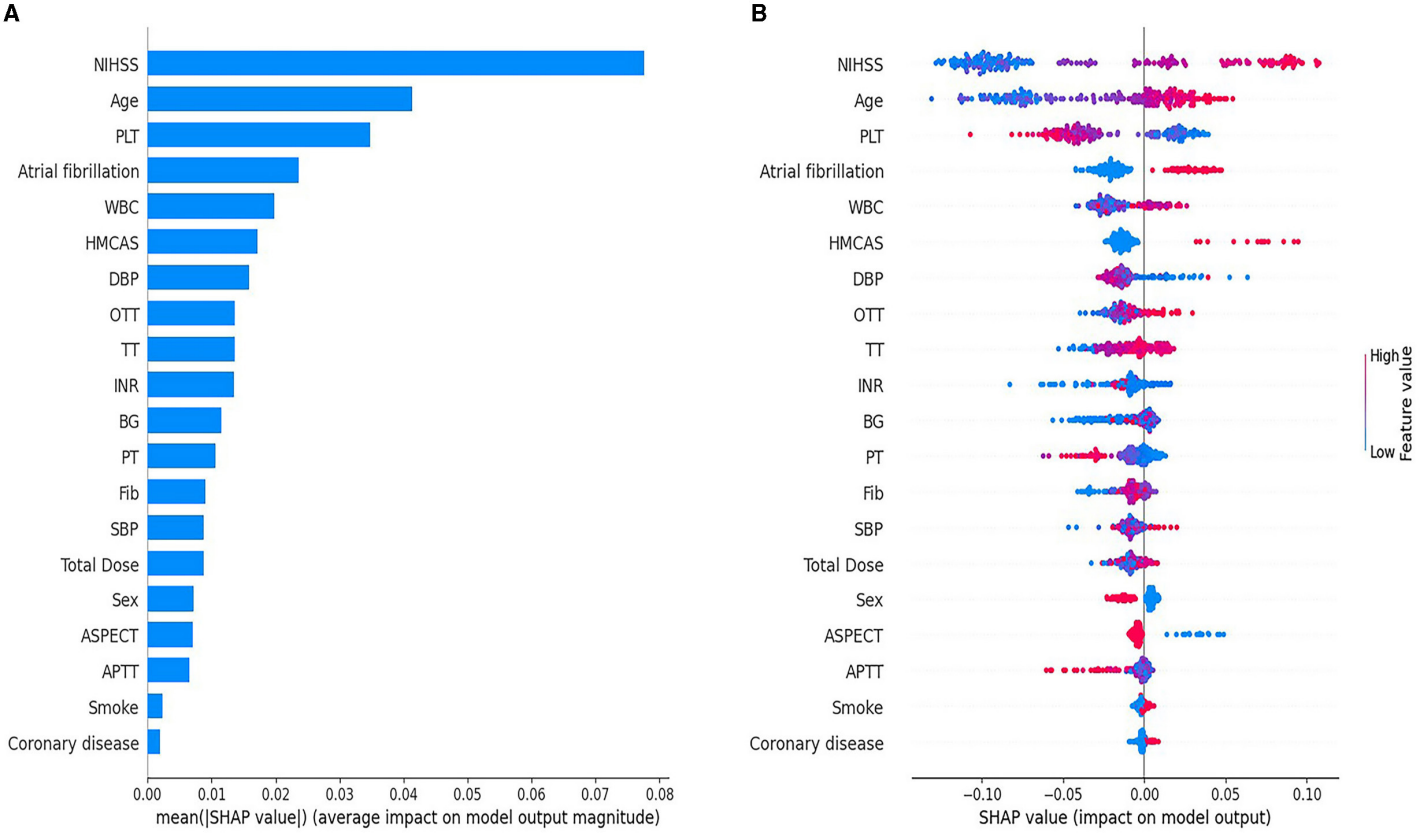
SHAP global feature plot **(A)** and SHAP summary plot **(B)** for the ML-HT model based on RF algorithm. **(A)** The SHAP global feature plot displays the average importance of the top 20 predictive variables in the model. Each bar corresponds to a predictive variable, and the bar's height signifies its global importance, ranked from the largest to the least. **(B)** SHAP summary plot shows the marginal contribution of the top 20 predictive variables in the model. The horizontal distribution of points along the X-axis corresponds the SHAP values of the predictive variables, indicating their marginal contributions to the model. The color of the points denotes the actual values, with red signifying high and blue signifying low values. A clear separation in the distribution of points suggests a consistent contribution to the model, whereas overlapping color distributions imply an ambiguous contribution. The Y-axis ranks the predictor variables by their contribution to the model, from the greatest to the least. SHAP, SHapley Additive exPlanations; ML, machine learning; HT, hemorrhagic transformation; RF, Random Forest; NIHSS, National Institute of Health Stroke Scale; PLT, platelet; WBC, white blood cell; HMCAS, hyperdense middle cerebral artery sign; DBP, diastolic blood pressure; OTT, onset-to-treatment time; TT, thrombin time; INR, international normalized ratio; BG, blood glucose; PT, prothrombin time; Fib, fibrinogen; SBP, systolic blood pressure; ASPECTS, Alberta Stroke Program Early CT Score; APTT, activated partial thromboplastin time.

SHAP global feature plot and SHAP summary plot showed the top 20 predictive variables of the ML-HT model based on RF algorithm, highlighting their overall impact on HT following IV-tPA thrombolysis (Figure 5). The NIHSS score had the most significant contribution, followed by age, PLT, atrial fibrillation, and WBC in the SHAP global feature plot (Figure 5A). However, the SHAP summary plot indicated that, with the exception of WBC, NIHSS score, age, PLT, atrial fibrillation, and HMCAS exhibited a relatively consistent contribution to the model, although HMCAS showed a more varied color distribution, suggesting a less uniform effect (Figure 5B). Consequently, the NIHSS score, age, PLT, and atrial fibrillation might be identified as the primary variables influencing HT after IV-tPA thrombolysis.

**Figure 6:**
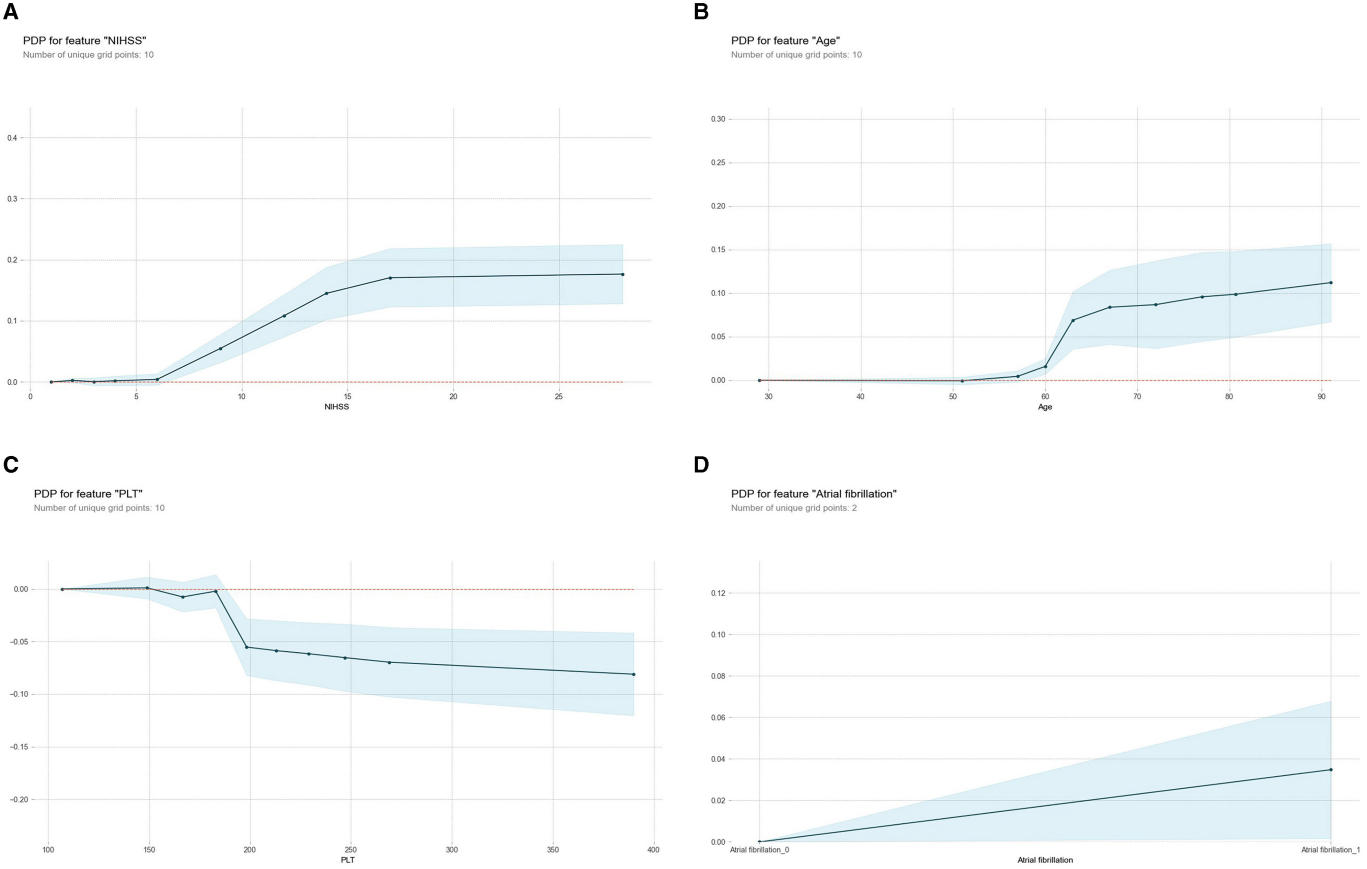
Partial Dependence Plot (PDP) for the ML-HT model based on RF algorithm. **(A)** PDP for feature “NIHSS;” **(B)** PDP for feature “age;” **(C)** PDP for feature “PLT;” **(D)** PDP for feature “atrial fibrillation.” The X-axis represents the range of values for each predictive variable, while the Y-axis indicates the probability of HT. Each PDP curve illustrates the trend of how probability of HT changes with respective predictive variable. The shaded area represents the 95% confidence interval, indicating the reliability of prediction. ML, machine learning; HT, hemorrhagic transformation; RF, Random Forest; PDP, Partial Dependence Plot; NIHSS, National Institute of Health Stroke Scale; PLT, platelet.

The PDP illustrated the influence of individual predictive variables on HT while holding all other variables constant, thereby highlighting the marginal contributions (Figure 6). Specifically, it showed the effects of the NIHSS score (Figure 6A), age (Figure 6B), PLT (Figure 6C), and atrial fibrillation (Figure 6D) to the likelihood of HT after IV-tPA thrombolysis. It is observed that the probability of HT increased progressively with a NIHSS score exceeding 6 points, age above 60 years, a PLT count below 180 × 10^9^/L, or a history of atrial fibrillation.

## 4 Discussion

In this study, we constructed four post-thrombolytic HT models based on different ML algorithms, including RF, MLP, AdaBoost, and GauNB. Initially, we assessed their predictive discrimination in original cohort and external independent cohort through stratified K-fold validation. The results indicated superior performance from the RF-based ML-HT model. Subsequently, we performed an explainable analysis of the model, revealing that the baseline NIHSS score, age, PLT, and atrial fibrillation were the primary factors contributing to HT after IV-tPA thrombolysis.

Unlike traditional statistical analysis methods, ML offers distinct advantages. ML algorithms can learn directly from data, balancing the impacts of various factors on target event, rather than relying on a few factors to determine the probability of that event. This capability enables the creation of more precise predictive models that exhibit strong generalization for unknown datasets. Moreover, as datasets are updated with samples of diverse distributions, ML models can continuously self-adjust and optimize, thereby enhancing the accuracy and stability of their predictions. Additionally, ML can reduce errors caused by human factors and mitigates selection bias (9). Several studies have proposed that integrating ML with clinical data to develop decision-making models can manage larger-scale, multi-dimensional data, while also enabling automated and visualized result outputs, which in turn boosts the efficiency of clinical workflows (10). In recent years, a limited number of studies have applied ML algorithms to predict different types of HT after intravenous thrombolysis, with quite promising outcomes (14–18).

Our study yielded several novel findings. Initially, we assessed the predictive performance of four ML algorithms, integrating their capabilities to select the most optimal ML-HT model. Additionally, all models employed stratified K-fold cross-validation to ensure robustness. Compared to existing predictive scores, the ML-HT models developed in this study incorporated data from 27 pre-thrombolysis predictive variables, thus minimizing selection bias. Lastly, we identified the ML-HT model with the superior performance and conducted explainable analysis to point out the primary factors.

NIHSS score is a reliable and sensitive scale for evaluating neurological deterioration, reflecting the severity of neurological impairment in AIS patients and demonstrating a good inter-rater consistency (19). Previous studies have utilized the baseline NIHSS score as a primary predictive factor for HT after IV-tPA thrombolysis, with higher baseline NIHSS scores indicating an elevated risk of HT (20–25). Consistent with previous studies, the explainable analysis of ML-HT model in present study showed that the baseline NIHSS score was a major factor for HT. Furthermore, PDP analysis demonstrated the marginal contribution of baseline NIHSS score to the ML-HT model, revealing that when the NIHSS score exceeded 6 points, its impact on the likelihood of HT after IV-tPA thrombolysis became more pronounced. In addition to the baseline NIHSS score, we frequently utilize early indicators from pre-thrombolysis imaging assessments to evaluate the severity of neurological deterioration in AIS patients. These early imaging indicators include the MCI sign (6), HMCAS (11), and ASPECTS score (12). They can reflect the extent of brain tissue ischemia and the compensatory capacity of collateral circulation (22, 23), and they have been confirmed by various studies as significant factors affecting HT following IV-tPA thrombolysis (4, 6, 12, 26–28). However, discrepancies in imaging interpretation among different observers have been noted, potentially impacting the reliability of these results (29, 30). The explainable analysis of the ML-HT model in present study showed that HMCAS was a relatively stable, but modestly contributing, factor influencing HT. This suggested that once the early signs of infarction from imaging are accurately assessed, they can be factored into the evaluation of HT risk post-thrombolysis.

Age has been established by numerous studies to be associated with HT after IV-tPA thrombolysis (20–23), possibly due to the reduced vascular elasticity, increased vascular fragility, diminished blood-brain barrier function, and alterations in coagulation mechanisms among the elderly (31). The European Cooperative Acute Stroke Study (ECASS)-III, which extended the treatment time window to 3–4.5 h after symptoms onset for AIS patients undergoing IV-tPA thrombolysis, specifically excluded patients over the age of 80, identifying advanced age as a risk factor for HT after thrombolysis (32). In explainable analysis of the ML-HT model in present study, age emerged as a significant contributor to the likelihood of HT. PDP analysis of the ML-HT model showed that when age exceeded 60, the incidence of HT would gradually increase. As the global population continues to age, an increasing number of elderly AIS patients will be candidates for IV-tPA thrombolysis. Given the findings of this study, it is recommended that elderly patients should be screened for thrombolysis according to the guidelines, taking into account both the selection criteria and the potential benefits of intravenous thrombolysis treatment.

Atrial fibrillation is one of the risk factors for ischemic stroke and is also a major influencing factor for HT after IV-tPA thrombolysis for AIS patients (20–23, 33, 34). Consistent with previous findings, the explainable analysis of the ML-HT model in present study showed that a history of atrial fibrillation played a crucial role in HT risk prediction of the ML-HT model. Atrial fibrillation may raise the risk of HT after intravenous thrombolysis through various mechanisms, including damaging the integrity of the blood-brain barrier, exacerbating ischemic vascular endothelial cell damage, increasing inflammatory responses, and enhancing capillary permeability. Furthermore, when atrial fibrillation results in cardioembolism, the larger ischemic area and inadequate collateral circulation can worsen reperfusion injury and other pathological changes, potentially leading to post-thrombolytic HT (35, 36). Despite atrial fibrillation elevating the risk of HT, it is essential to weigh the potential benefits and risk of intravenous thrombolysis for patients with this condition.

Platelet count is an indicator for assessing coagulation function, participating in thrombus formation in the coagulation process. A low platelet count may increase bleeding tendency and may consequently elevate the risk of HT after IV-tPA thrombolysis. Currently, in the treatment guidelines for AIS patients, platelet count is considered a potential risk factor for HT after intravenous thrombolysis. However, whether to regard platelet count as an independent risk factor is still unclear (19, 37). In explainable analysis of our ML-HT model, platelet count emerged as one of the key decision-making factors influencing the risk of HT after intravenous thrombolysis, aligning with the findings from the MSS (5). Nonetheless, in clinical practice, it is imperative to consider a comprehensive range of coagulation-related factors, including platelet function, the patient's history of antiplatelet and anticoagulant medication use, and any history of hematological disorders. Platelet count alone should not be the sole determinant in assessing risk. The ML-HT model can be instrumental in decision-making, providing a more nuanced assessment of the risk for HT after IV-tPA thrombolysis.

There are several limitations in the present study. First, our findings were limited by the nature of the retrospective study, with the potential for recall bias. Second, the single-center design of our research may restrict the generalizability of our results. Therefore, further validation is still needed in prospective, multi-center cohorts. Lastly, although our ML-HT model was semi-automated, because the imaging data still required manual interpretation, which could introduce inter-observer variability. Future enhancements could potentially increase the model's automation by integrating deep learning algorithms, renowned for their powerful imaging recognition capabilities.

## 5 Conclusion

In this study, we developed and validated an accurate ML-HT model for predicting HT following IV-tPA thrombolysis based on RF algorithm. The explainable analysis of the model revealed that the baseline NIHSS score, age, PLT, and atrial fibrillation were the primary factors contributing to HT.

## Data Availability

The raw data supporting the conclusions of this article will be made available by the authors, without undue reservation.
